# Case presentation: Very early combined first-line immunotherapy and surgery in tumor-associated anti N-methyl-d-aspartate (NMDA) receptor encephalitis associated with improved outcome

**DOI:** 10.1192/j.eurpsy.2023.786

**Published:** 2023-07-19

**Authors:** S. Witzel, R. Zeiss

**Affiliations:** 1 Department of Neurology; 2 Department of Psychiatry, Ulm University, Ulm, Germany

## Abstract

**Introduction:**

Anti N-methyl-d-aspartate receptor (anti-NMDA-R) encephalitis is a potentially reversible cause of psychosis. Nearly all patients (>95.5%) quickly develop additional neurological symptoms, and only about 50 percent fully recover, often with latency. At symptom onset, patients commonly present with isolated psychosis, making it challenging to distinguish the disease from a primary psychiatric disorder: The median time from symptom onset to immunotherapy in adults is 28 days (IQR 14–49), and from symptom onset to surgery in tumor-associated cases 1.4 months (IQR 0.7–2.6). Steroids, intravenous immunoglobulins (IVIG), or plasmapheresis are recommended as first-line immunotherapies.

**Objectives:**

To highlight a case with improved outcome after very early combined first-line immunotherapy (steroids plus IVIG) and surgery in tumor-associated anti-NMDA-R encephalitis.

**Methods:**

Workup of the clinical case followed by a review of the literature.

**Results:**

We present the case of a 33-year-old woman with sudden onset of anxiety and jealousy ideas, which within a few days, developed a manifest psychosis with formal thought disorders, paranoid and guilt delusions, distrust, and orientation disorders in the absence of additional neurological deficits. A lower abdominal tumor suspicious of an ovarian tumor was detected sonographically five days before the onset of the first symptoms. Lumbar puncture and abdominal computer tomography were performed within 30 hours after hospital admission, confirming the diagnosis of tumor-associated anti-NMDA-R encephalitis with autoantibodies in CSF and serum at a very early clinical stage. First-line immunotherapy with steroids (methylprednisolone 1000mg, day 6 to 10 after symptom onset) was started immediately and combined with IVIG therapy (0.35g/kg, day 9 to 13 after symptom onset). Surgery of the ovarian tumor was performed on day 14 after admission, with histology revealing an immature teratoma. The neuropsychatric examination on day 1 after surgery showed complete remission of clinical symptoms, which persisted during clinical follow-up after 1 and 4 months.

**Conclusions:**

The present case highlights the role of early CSF diagnostic and tumor assessment if autoimmune encephalitis is suspected. Very early first-line immunotherapy with steroids and IVIG, complemented by tumor surgery, was associated with improved outcome in this case with anti-NMDA-R encephalitis. Further studies are warranted to evaluate the generalizability of the finding.

**Disclosure of Interest:**

None Declared

